# Selective Laser Trabeculoplasty versus Argon Laser Trabeculoplasty in Patients with Open-Angle Glaucoma: A Systematic Review and Meta-Analysis

**DOI:** 10.1371/journal.pone.0084270

**Published:** 2013-12-19

**Authors:** Wei Wang, Miao He, Minwen Zhou, Xiulan Zhang

**Affiliations:** Zhongshan Ophthalmic Center, State Key Laboratory of Ophthalmology, Sun Yat-sen University, Guangzhou, People’s Republic of China; Massachusetts Eye & Ear Infirmary, Harvard Medical School, United States of America

## Abstract

**Objective:**

To examine possible differences in clinical outcomes between selective laser trabeculoplasty (SLT) and argon laser trabeculoplasty (ALT) in open-angle glaucoma at different times post-treatment.

**Methods:**

Randomized controlled trials (RCTs) comparing SLT versus ALT were searched through August 2013. The main outcome measure was IOP, and secondary outcomes included the number of glaucoma medications, the success rate, and adverse events.

**Results:**

Six RCTs, involving 482 eyes treated with laser trabeculoplasty, were included in the meta-analysis. For all patients (including first and previous laser trabeculoplasy), no significant difference in IOP lowering was observed between SLT and ALT at one hour (P = 0.40), one week (P = 0.72), one month (P = 0.37), six months (P = 0.08), one year (P = 0.34), two years (P = 0.58), three years (P = 0.34), four years (P = 0.47), and five years (P = 0.50). A statistically significant difference in favor of SLT was found when comparing the IOP reduction at three months after intervention (weighted mean difference (WMD): 1.19 mmHg [0.41; 1.97]; I^2^=0%; P = 0.003). For patients who were naive to laser, there was no significant difference of reduction in IOP comparing SLT with ALT at any time point. In patients’ previous LT, no statistically significant difference in IOP reduction was found at six months (WMD: 1.92 mmHg [-0.91; 4.74]; I^2^ = 77.3%; P = 0.18). There was no significant difference in the reduction in the number of glaucoma medications, the success rate, or adverse event rates between the two treatments.

**Conclusions:**

SLT has equivalent efﬁcacy to ALT with a similar constellation of side effects. In the case of retreatment, SLT appears to be similar to ALT in IOP lowering at six months.

## Introduction

Glaucoma is a leading cause of irreversible blindness around the world. It is estimated that 8.4 million people will become blind from primary glaucoma by 2010, with this number rising to 11.1 million by 2020[1]. Lowering the IOP is still the most effective way to prevent the development and progression of glaucoma[1]. Currently, there are three methods available to achieve this goal: medical treatment, laser therapy, and surgical intervention[2].

Argon laser trabeculoplasty (ALT), introduced in 1979 by Wise and Witter, rapidly became a standard option in the clinical management of open-angle glaucoma (OAG)[3]. The five-year success rate with ALT is reported to be 50%, with a decrease of 6% to 10% per year[4]. However, ALT also had some side effects postoperatively, and histopathologic studies have revealed damage of the trabecular meshwork, which may limit retreatment with ALT. Selective laser trabeculoplasty (SLT) was developed by Latina in 1995 and FDA-approved in 2001, which provided a new choice for OAG[5,6]. This method uses a frequency-doubled, Q-switched Nd:YAG laser rather than an argon wavelength. It selectively ablates pigmented trabecular meshwork cells, minimizing thermal damage to adjacent cells and structures. 

Many published clinical trials have compared the efficacy and safety of SLT versus ALT[7–17]. However, these studies had modest sample sizes and conveyed inconclusive results[18,19]. In 2011, a report by the American Academy of Ophthalmology concluded that it remains unclear whether the theoretical advantages that the newer lasers offer can be translated into actual clinical advantage, and more evidence is necessary to determine whether they are equivalent [20]. Since this review, there have been further publications on this topic[21–25]. However, a quantitative assessment of all published randomized clinical trials (RCTs) is not available. Therefore, we conducted a systematic review and meta-analysis of RCTs to assess the efficacy and tolerability of both procedures in the treatment of OAG.

## Materials and Methods

The Preferred Reporting Items for Systematic Reviews and Meta-Analyses (PRISMA) statement was used as a guide to conduct the study, including the strategies for searching, analysis, and the presentation of results, potential bias, interpretation, and writing [26]. 

### 1: Literature search and inclusion criteria

RCTs were identiﬁed through a systematic search of PubMed, Embase, the Web of Science, the Chinese Biomedicine Database, and the Cochrane Controlled Trials Register up to August 2013. The structured search strategies used the following format for search terms: (“selective laser trabeculoplasty” or “selective laser trabeculectomy” or “Nd:YAG” or “SLT”) *and* (“argon laser trabeculoplasty” or “argon laser trabeculectomy” or “ALT”). No restriction was applied for language or year of publication. The websites of professional associations and Google Scholar were also searched for additional information. Moreover, a manual search was performed by checking the reference lists of all retrieved trials to identify studies not yet included in the computerized databases. Eligible studies were prospective randomized clinical trials comparing the use of ALT and SLT in adult patients with any form of open-angle glaucoma. 

### 2: Data extraction and outcome measures

Selection, data collection, and assessment of the methodological quality of the studies were conducted independently by two reviewers (W.W. and MW.Z.) in a standardized way. Any disagreement was resolved by discussion. For each study and each type of treatment, the following data were extracted: first author, publication year, information on study design, location of the trial, duration of the study, number of subjects, age, sex, type of glaucoma, IOP measurements, and other important clinical outcome data. The numbers of withdrawals and patients reporting adverse events were also recorded. For the publications reporting on the same study population, the article reporting the results of the last endpoint was included, and data that could not be obtained from this publication were obtained from others. The primary outcome was the intraocular pressure (IOP) reduction at different times post-treatment. Secondary outcomes included the number of glaucoma medications, the success rate, and adverse event rates. 

### 3: Quality and risk-of-bias assessment

The quality assessment was performed according to the risk-of-bias tool outlined in the *Cochrane Handbook for Systematic Reviews of Interventions* (version 5.1.0)[27]. Six different key aspects that inﬂuence the quality of an RCT were assessed: sequence generation, allocation concealment, blinding of patients, personnel and outcome assessors, management of eventual incomplete outcome data, completeness of outcome reporting, and other potential threats to validity. 

### 4: Statistical analysis

All analyses were performed on an intent-to-treat basis (i.e. all patients assigned randomly to a treatment group were included in the analyses according to the assigned treatment irrespective of whether they received treatment or were excluded from analysis by the study investigators). The weighted mean difference (WMD) was calculated for continuous outcomes. For dichotomous outcomes, the relative risk (RR) was estimated. All results were given with 95% conﬁdence intervals (CIs). Heterogeneity was checked using Cochran's Q statistic and the P-value. I^2^ metrics, which quantify heterogeneity irrespective of the number of studies, were also reported. Studies with an I^2^ value of greater than 50% exhibit significant heterogeneity[28]. The analysis of efficacy data was stratified by the duration of follow-up. Subgroup analysis was performed according to whether the patients were naive to laser. We also investigated the influence of a single study on the overall pooled estimate by omitting one study in each turn. To detect publication biases, we explored asymmetry in funnel plots. These were examined visually; furthermore, the Egger measure of publication bias was calculated[29]. A P-value of less than 0.05 was considered statistically significant. The statistical analysis was performed using Stata version 12.0 (Stata Corporation LP, College Station, TX, USA) according to the method described by DerSimonian and Laird[30].

## Results

### 1: Study identification and selection

**Figure 1:**
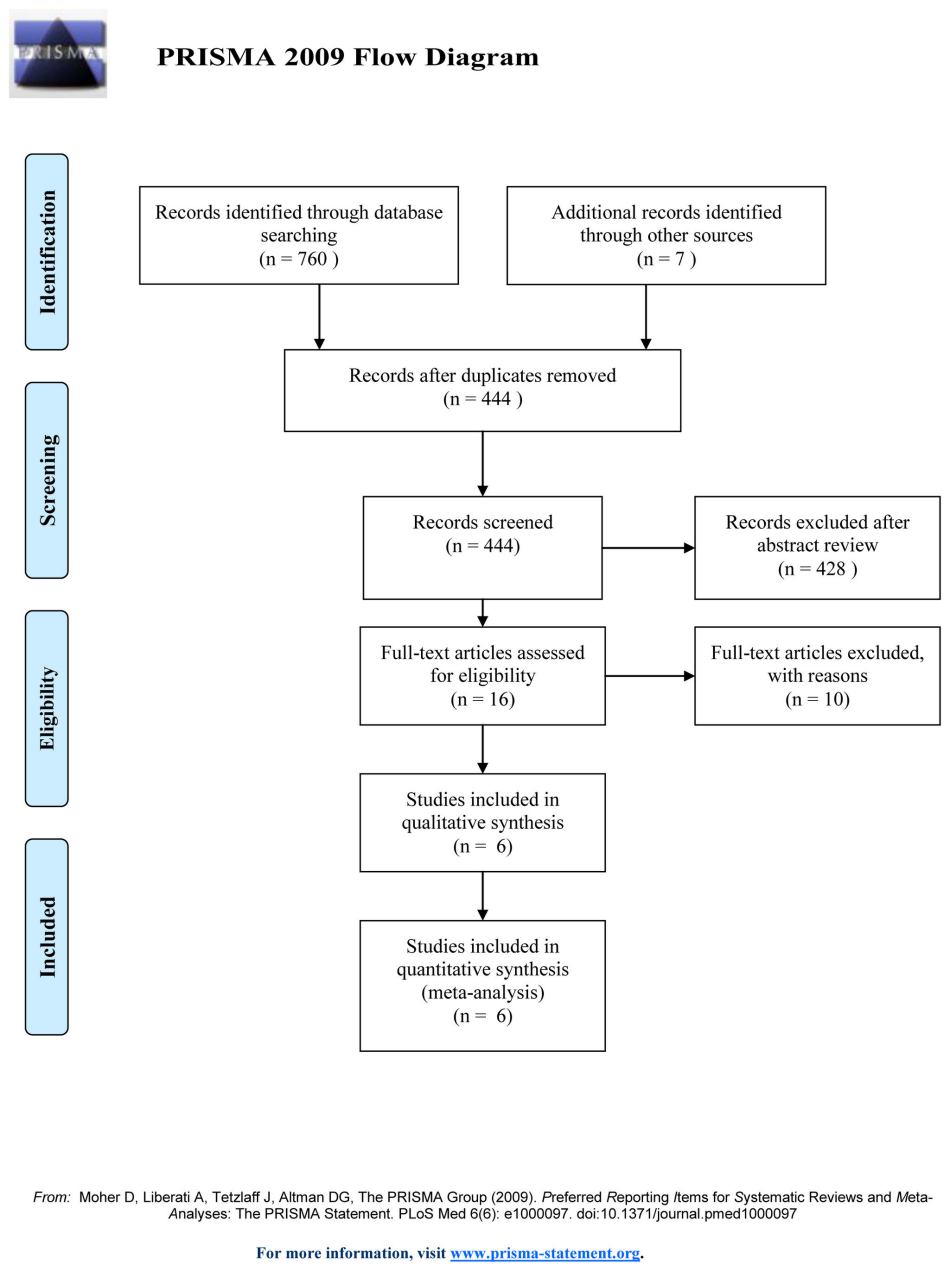
Selection process for randomized controlled trials included in the meta-analysis.

The initial search yielded 767 relevant publications, of which 751 were excluded for duplicate studies and various reasons (reviews, case series, SLT versus drugs rather than ALT, or irrelevant to our analysis) on the basis of the titles and abstracts. The remaining 16 were retrieved for full-text review, and 10 of them were excluded because one was a quasi-RCT study[7], four were non-RCTstudies[11,13,17,25], two provided inadequate data[9,15], and three contained duplicated data[10,12,16]. Thus, six RCTs [8,14,21–24] were included in the final analysis. In one trial[8], two kinds of patients who received SLT were involved, 27 subjects (group A) were treated with SLT after previously receiving 360-degree ALT therapy; 30 patients (group B) were given their first laser treatment. We assumed group A and group B to be two separate studies. The trial selection process is shown in Figure 1.

### 2: Study characteristics

The characteristics of RCTs included in the current meta-analysis are presented in Table 1, and the treatment settings of each treatment are described in Table 2. Overall, 442 patients (482 eyes) were evaluated for an average period of follow-up ranging from 3.1 to 60 months. Two hundred fifty-three eyes were treated with SLT and 229 with ALT. The patients' mean age ranged from 48.7 to 73.4 years. Among all the eyes, 45.85% belonged to males and 54.00% belonged to females. Of the six trials that were included in this meta-analysis, four were done in Canada[8,21,23,24], one in Israel[22], and one in Spain[14]. The types of OAG include POAG, PXFG, and mixed. SLT is performed at 180° with a mean power of 0.7-1.2 mJ and 45–70 applications. ALT is usually performed using 45 55 applications of a 50 μm spot size and an average power setting of between 400 and 850 mW.

**Table 1:** Characteristics of randomized controlled trials comparing SLT versus ALT.

Author(year)	Group	Eye	Patients	Follow-up(m)	Age	Sex(M/F)	Type of Glaucoma*	Previous ALT	Baseline IOP	No of glaucoma medications:
Birt(2007)	SLT-A	30	30	12	64.0	15/15	25/3/2	0	22.9 ±4.2	2.9 ± 1.2
	SLT-B	27	27	12	72.4	14/13	22/4/1	27	21.5 ± 4.3	2.8 ± 1.2
	ALT	39	39	12	70.0	21/18	34/5/0	0	22.0 ±5.3	2.8 ± 1.2
Bovell(2011)	SLT	89	152	60	69.7	36/53	54 /23/12	28/61	23.8 ± 4.9	2.6 ± 1.20
	ALT	87		60	69.5	36/51	48/29/9	39/48	23.5 ± 4.2	2.4 ± 1.24
Liu(2012)	SLT	20	20	3.1	48.7	15/5	9/0/11	0	19.1±4.5	2.6 ± 0.9
	ALT	22	22	3.2	51.6	14/8	10/0/12	0	21.9±4.4	2.9 ± 0.8
Rosenfeld(2012)	SLT	22	22	12	71.95	11/11	9/7/6	0	25.4±1.8	NA
	ALT	30	30	12	71.9	14/16	14/8/8	1	25.1±2.2	NA
Casa(2004)	SLT	20	20	6	73.4	9/11	20/0/0	0	24.0±4.7	1.8±0.5
	ALT	20	20	6	72.5	10/10	20/0/0	0	23.6±3.8	1.5±0.7
Kent(2013)	SLT	45	60	6	72.9	16/29	0/45/0	0	23.1 ± 4.2	NA
	ALT	31		6	73	10/21	0/31/0	0	25.2 ± 4.9	NA

* POAG/PXFG/Other type of OAG; Abbreviations: SLT = selective laser trabeculoplasty ; ALT =argon laser trabeculoplasty; IOP= intraocular pressure; M/F=male/female; m=month; NA: not available.

**Table 2:** Summary of treatment settings for SLT and ALT in the management of open angle glaucoma.

Author(year)	SLT					ALT			
	Degrees treated	Mean power (mJ)	Totoal energy	Number of spots		Degrees treated	Mean power (mW)	Totoal energy	Number of spots
Birt(2007)	180	0.7 or 0.8	NA	45 to 55		180	700 and 850	NA	45 to 55
Bovell(2011)	180	0.8	NA	50		180	550	NA	50
Liu(2012)	180	0.7 to 0.8	NA	45 and 55		180	500 to 800	NA	45 and 55
Rosenfeld(2012)	180	0.8 to1.2	NA	50 to 70		180	400 to 600	NA	50
Casa(2004)	180	0.9	48.3	52.3		180	768.9	4321	56.2
Kent(2013)	180	NA	31.9	53		180	NA	632.2	51

Abbreviations: SLT = selective laser trabeculoplasty ; ALT =argon laser trabeculoplasty; NA: not available.

### 3: Quality and bias assessment of studies

To address the strength of evidence in this study, we evaluated the risk of bias of the six RCTs (Table 3). In general, the included trials were of good quality for most of the aspects evaluated. Sequence generation was appropriate in all studies except in two studies, where the method was unclear. Allocation concealment was described in two studies. In the other four studies, it was unclear. Three studies were not appropriately masked because of the impracticality of masking patients to laser trabeculoplasty. Five studies used an intention-to-treat method. Only one study failed to address incomplete data outcomes. All studies were judged to be of low risk of bias from selective reporting because it was clear from the published articles that all main pre-specified outcomes were reported.

**Table 3:** Results of Cochrane collaboration’s tool of assessing of bias.

Trial (year)	Sequence Generation	Allocation Concealment	Blinding			Adequate asseement of each outcome	Selective reporting avoided	No Other Bias
			Patient	Personnel	Assessor			
Birt(2007)	Yes	Unclear	No	No	No	Yes	Yes	Yes
Bovell(2011)	Yes	Yes	Unclear	No	No	Yes	Yes	Yes
Liu(2012)	Unclear	Unclear	No	No	No	Yes	Yes	Yes
Rosenfeld(2012)	Yes	Yes	Unclear	Unclear	Unclear	No	Yes	Yes
Casa(2004)	Unclear	Unclear	Unclear	Unclear	Unclear	Yes	Yes	Yes
Kent(2013)	Yes	Unclear	No	No	No	Yes	Yes	Yes

No=category was not addressed adequately; Yes=category was addressed adequately; Unclear = insufficient information to permit judgment of Yes or No.

### 4: Primary outcome: IOP reduction

For all patients (including first and previous laser trabeculoplasty), there was no statistically significant difference in the amount of IOP reduction between SLT and ALT at all intervals, with the exception of three months (Table 4). When comparing the IOP reduction three months after intervention, a statistically significant difference in favor of SLT was found [weighted mean difference (WMD): 1.19 mmHg (0.41; 1.97); I^2^ = 0%; P = 0.003]. However, no significant difference was observed between the two treatments at one hour (P = 0.40), one week (P = 0.72), one month (P = 0.38), six months (P = 0.08), one year (P = 0.34), two years (P = 0.58), three years (P = 0.34), four years (P = 0.47), and five years (P = 0.50).

**Table 4:** Pooled estimates for intraocular pressure and glaucoma medication reduction from baseline for SLT versus ALT.

Index	Follow-up	No. of studies	SLT		ALT		WMD(95%CI)		Heterogeneity				Overall effect	
			Estimate (95%CI)		Estimate (95%CI)				Q	P	I2		Z	P
IOP(mmHg) (All patients)	1h	4	1.38(-0.19,2.95)		1.89(-1.37,5.15)		-0.80(-2.65,1.06)		9.68	0.021	69.00%		0.84	0.40
	1w	4	3.88(2.19,5.57)		3.48(1.83,5.13)		0.24(-1.07,1.55)		6.62	0.09	54.70%		0.36	0.72
	1m	4	4.76(3.69,5.84)		3.92(2.57,5.27)		0.50(-0.59,1.59)		5.21	0.16	42.40%		0.90	0.37
	3m	4	4.79(4.01,5.57)		3.29(2.58,4.00)		1.19(0.41,1.97)		2.29	0.51	0.00%		3.00	0.003
	6m	6	7.36(2.56,12.15)		6.94(1.35,12.54)		0.67(-0.07,1.42)		1.77	0.88	0.00%		1.78	0.08
	1y	4	4.86(4.12,5.60)		4.63(3.28,5.97)		0.37(-0.38,1.12)		3.83	0.43	0.00%		0.96	0.34
	2y	2	3.73(0.21,7.25)		4.41(2.20,6.62)		-0.43(-1.95,1.08)		0.32	0.57	0.00%		0.56	0.58
	3y	1	6.80(5.19,8.41)		5.90(4.67,7.13)		0.90(-0.96,2.76)		-	-	-		0.95	0.34
	4y	1	7.30(5.69,8.91)		6.40(5.25,7.55)		0.70(-1.21,2.61)		-	-	-		0.72	0.47
	5y	1	7.90(6.42,9.38)		6.60(5.10,8.11)		0.70(-1.36,2.76)		-	-	-		0.67	0.50
No. of medications	Endpoint	4	0.55(-0.02,1.12)		0.01(-0.17,0.19)		0.57(0.00,1.14)		14	0.003	78.60%		1.96	0.05
IOP(mmHg) (naive to laser)	1h	2	0.72(-4.17,5.62)		1.56(-7.46,10.57)		-1.00(-5.12,3.11)		4.64	0.031	78.50%		0.48	0.63
	1w	2	4.22(-0.09,8.52)		4.78(0.67,8.90)		-0.95(-2.69,0.78)		0.03	0.87	0.00%		1.07	0.28
	1m	2	5.76(3.99,7.52)		4.98(1.84,8.11)		0.01(-1.94,1.97)		1.37	0.24	26.80%		0.01	0.10
	3m	2	5.23(3.66,6.81)		3.71(2.18,5.24)		1.48(-0.10,3.06)		0.01	0.90	0.00%		1.83	0.07
	6m	3	5.99(4.79,7.20)		5.09(3.31,6.87)		0.70(-0.70,2.10)		1.58	0.46	0.00%		0.98	0.33
	1y	2	4.65(3.08,6.23)		4.31(1.48,7.13)		0.11(-1.51,1.73)		0.66	0.42	0.00%		0.13	0.90
	2y	1	1.80(-0.75,4.35)		2.80(-0.21,5.81)		-1.00(-3.50,1.50)		-	-	-		0.78	0.43

Abbreviations: SLT = selective laser trabeculoplasty ; ALT =argon laser trabeculoplasty; IOP= intraocular pressure;

We divided the studies into two groups according to whether the patients received previous failed laser treatment. For patients who were naive to laser (first laser trabeculoplasty), there was no significant difference of IOP reduction when comparing SLT with ALT at any time point (all P > 0.05) (Table 4). In patients who had received previous laser treatment, the difference in IOP reduction was also statistically non-significant at six months after the retreatment (WMD: 1.92 mmHg [-0.91; 4.74]; I^2^ = 77.3%; P = 0.18). The result revealed that SLT is equally effective in IOP lowering compared with ALT. However, we could not compare IOP reduction for patients who experienced previous laser treatment between SLT and ALT at other time points because of a lack of studies on this topic.

### 5: Secondary outcomes

Four trials reported the number of glaucoma medications that the patients took before and after laser treatment. The pooled results showed no signiﬁcant difference in glaucoma medication reduction between the two groups (Table 4). Studies by Bovell, Liu, Rosenfeld, and Casa compared the success rate between the two treatment groups at the last follow-up visit. No significant difference in success rate was found with RR (95% CI) of 1.03 (0.83, 1.28).

Concerning adverse events, three trials[8,22,24] reported the proportions of eyes requiring laser retreatment, trabeculectomy or other procedure to lower IOP within one year, no differences were found between SLT and ALT, with RR (95% CI) of 0.40 (0.16, 1.01), 0.99 (0.30, 3.24), and 2.48 (0.10, 64.74), respectively. As for anterior inflammation after laser, one study[24] reported that SLT was associated with a significantly higher number of cells in the anterior chamber, while another study[14] found lower anterior chamber flare in the SLT group during the initial postoperative hours. However, we did not perform a meta-analysis because the reports lacked a uniform standard of measuring postoperative inflammation in the anterior chamber. One study compared the incidence of IOP spike between the two treatments, and its rate was also similar for SLT and ALT. 

### 6: Sensitivity analysis and publication bias

To analyze the consistency and robustness of the results, each study was excluded one at a time and the analysis performed again to compare with the previous analysis. None of the clinical trials included in this meta-analysis had an important impact on the global estimation of the IOP reduction, suggesting high stability of the meta-analysis results (data not shown). A funnel plot based on IOP reduction at six months was created. The relatively symmetrical distribution suggests the absence of publication bias despite the small number of trials that were included in this meta-analysis.

## Discussion

In this meta-analysis, we reviewed six randomized clinical trials, and the results reveal that SLT is nearly as effective as ALT in regard to the control of IOP, which is consistent with another earlier review [3,20]. For all patients, only the differences at three months reached a level of significance. SLT and ALT are similar in IOP lowering in patients without a prior laser treatment over the five-year period. In the case of retreatment, SLT appears to be equivalent to ALT in IOP lowering at six months. However, whether SLT has better long-term success than ALT in repeat laser trabeculoplasty treatments remains unclear. In addition, SLT and ALT are similar in their success rates, glaucoma medication reduction, and complication rates. 

Several non-randomized studies[7,11,13,17,25] comparing SLT with ALT are summarized in Table 5. All of them reported that SLT and ALT are similar in their biological effects, complication rates, and capabilities in their IOP reduction potential among the investigated patient groups. The principal finding of the aforementioned studies on the topic seems to be consistent with the present meta-analysis. However, the limitations of these studies are that a non-randomized study design was used (case control or pre/post-intervention observational study). 

**Table 5:** Summary of non-randomized studies comparing SLT with ALT.

Tial(year)	Design	No.eyes	Previous therapy	Follow-up	Outcome measure	Result		P
						SLT	ALT	
Almeida(2011)	Prospective, non-RCT	45	MMT	6m	Percent IOP reduction	7d 23.7%	8.1%	< 0.001
						1d, 1, 3, 6m		≥ 0.32
Russo (2009)	Prospective non-RCT*	120	MMT	12m	Percent IOP reduction	26.5%	26.6%	>0.05
Van de Veire (2006)	Retrospective	56	Topical medications	3-5w	Percent IOP reduction	15.5%	22.4%	0.14
Juzych(2004)	Retrospective	195	MMT±prior ALT	37.4m(SLT)33.6m(ALT)	Percent with ≥3mmHg reduction without further therapy	1y:58% 3y:38% 5y:32%	1y:54% 3y:30% 5y:31%	0.20
					Percent with ≥20% reduction without further therapy	1y:68% 3y:46% 5y:31%	1y:46% 3y:23% 5y:13%	0.12
Hollo(1996)	Prospective, non-RCT	14	MMT	18m	IOP reduction	NA	NA	>0.05

* quasi-RCT (based on clinic chart number); Abbreviations: ALT =argon laser trabeculoplasty; IOP= intraocular pressure; MMT=maximal medical therapy; SLT = selective laser trabeculoplasty

SLT has been in use for more than a decade, but very few long-term prospective studies appear to be available concerning its safety and efﬁcacy[31]. In our study, SLT was found to be as effective as ALT in lowering IOP over a five-year period. However, there was a possibility of bias in the efficacy of SLT versus ALT because of the use of additional interventions in many included trials. Many patients have received ALT during their clinical management. A retreatment is a second application of laser to meshwork that has previously received therapy[32]. The patients who have completed 360° of previous laser trabeculoplasty with the argon laser can still benefit from selective laser treatment, showing decreases in IOP very similar to those of patients who were naïve to prior laser therapy. Damji et al.[10] included some patients who had previously received ALT and found in a post-hoc analysis that patients with previous failed ALT or SLT had a better outcome when treated with SLT vs. ALT. When we compared the group of patients that had previously received ALT treatments, we found that there was no signiﬁcant difference in IOP lowering at six months between the SLT and ALT groups. However, whether SLT has better long-term efficacy than ALT in repeat laser trabeculoplasty treatments remains unclear.

Our meta-analysis showed that SLT also did not differ with ALT with respect to other important clinical outcomes, including the number of glaucoma medications and the success rate. These results are not conclusive, as further adequately powered studies are needed. In fact, these included studies are not sufficient to examine these secondary outcome measures since they were not the primary outcomes and were the only clinically significant endpoints consistently reported in many of the studies analyzed in the present meta-analysis. As far as side effects are concerned, both techniques are generally well tolerated ,with few complications. However, the limited follow-up and sample size do not allow a definite conclusion about the long-term complications of these procedures. The promise of reducing injury to the trabecular meshwork with SLT is a potential advantage but remains theoretical[33]. Further studies should pay more attention to these clinical endpoints other than just the IOP reduction.

Our study provides additional interesting clues that may be useful for future research on the topic. Remarkably, the study conducted by Casa et al.[14] included in our meta-analysis suggested SLT were associated less pain and inflammation. In addition to assessing IOP lowering, they compared postoperative pain and anterior chamber inﬂammation and found no differences in IOP lowering, but the group receiving ALT reported more pain and demonstrated more anterior chamber inﬂammation compared with the SLT group. Thus, one may focus on this specific outcome to address better the mechanical difference underlying SLT and ALT. More large-scale and well-performed RCTs are warranted.

While the current study was in progress, Wang et al. reported a small meta-analysis[34]. They analyzed data from six comparative studies and reported that SLT was associated with a relatively higher efﬁcacy of IOP lowering and a larger reduction in the number of glaucoma medications compared with ALT. The authors also found that SLT was more effective in IPR in patients who had not responded favorably to previous laser treatment, and patients who received SLT needed fewer glaucoma medications than those who received ALT. However, no difference in efficacy was found in the present meta-analysis. Some speciﬁc points may explain the discrepant ﬁndings, which are considered weak points in the former analysis. Studies of lower evidence level were included in that meta-analysis, including one prospective non-randomized trial[25] and one quasi-RCT[7]. In addition, the previous meta-analyses did not separate the studies by the length of follow-up, which can influence the study results. In the current meta-analysis, only high-quality RCTs were included, and a wider range of clinically relevant outcome measures were used; we also stratified the analysis of efficacy data by duration of follow-up using a rigorous statistical method. Of note, we added the latest two RCTs[21,22], involving 128 eyes, to increase the sample size and improve test performance. Unlike that described by Wang et al., SLT was associated with equivalent efficacy in IOP lowering and medication reduction compared with ALT. 

Some limitations of this meta-analysis should be taken into account. First, our analysis is based on only six RCTs, and some of them were carried out with small or very small sample size, inadequate allocation concealment, or inadequate or no double blinding. These factors may have a potential impact on our results. Second, the criteria used to deﬁne success vary between studies. Although the above assessments are widely used as outcome measures in clinical trials, further research is still needed to determine fully their validity, reliability, and sensitivity to changes. Third, several pooled data sets are based on only a few papers, especially IOPRs beyond three years, and more research is needed on the available guidance derived from the current literature. Fourth, publication bias cannot be fully excluded because, without sufficient studies, the Begg and Egger tests have low power to detect publication bias[35]. To avoid publication bias, both electronic and manual searches were conducted to identify all potentially relevant articles. Fifth, because of lack of patient stratiﬁcation into different types of OAG, our ﬁndings may not be extrapolated to other forms of glaucoma[36]. Finally, most of the population included here are whites, so the conclusion may not be true for other races and areas. All these limitations point toward the direction for future studies.

## Conclusions

Despite its various limitations, our study is still clinically valuable because it suggests that SLT has at least comparable efﬁcacy to ALT with a similar constellation of side effects. However, relevant evidence concerning whether SLT has improved long-term efficacy compared to ALT in the retreatment of patients with trabeculoplasty is still limited but accumulating. Thus, further large-scale, well-designed RCTs are urgently needed.

## Supporting Information

Table S1 **PRISMA checklist.**
(DOC)
